# The impact of expanding health insurance coverage for anti‐cancer drugs on cancer survival in Korea

**DOI:** 10.1002/cam4.3979

**Published:** 2021-06-18

**Authors:** Do‐Yeon Cho, Juhee Park, Dong‐Sook Kim

**Affiliations:** ^1^ Department of Research Health Insurance Review & Assessment Service Wonju South Korea

**Keywords:** all‐cause mortality, cancer, health insurance, overall survival, treatment outcomes

## Abstract

**Background:**

To reduce out‐of‐pocket costs, the Korean government expanded health insurance reimbursement in anti‐cancer drugs for cancer patients in 2013. Our objective was to examine the impact of the benefit coverage expansion policy on healthcare utilization and overall survival (OS) among patients with six types of solid cancer after the policy of expanding health insurance coverage.

**Methods:**

This study analyzed a before‐and‐after retrospective cohort of patients newly diagnosed with six types of solid cancer (stomach cancer, colorectal cancer, lung cancer, liver cancer, breast cancer, and prostate cancer) from January 1, 2009 to December 31, 2015 in Korea. The intervention was the expansion of reimbursement in 2013. Multivariate Cox proportional hazards regression was used to estimate the policy effect.

**Results:**

In total, 142,579 before and 147,760 patients after the benefit expansion, and after matched by age, gender, and stage, 132,440 before and 132,440 patients after policy were included in the analysis. Almost total medical expenditure increased for five types of cancer increased. The expansion of health insurance reimbursement was associated with significantly lower overall mortality compared with pre‐policy mortality for all six cancer sites.

**Conclusion:**

The policy of expanding health insurance reimbursement might have been associated with a significant increase in survival among cancer patients by ensuring access to health care and medicine. Although the reimbursement expansion timing differs for each cancer, it is believed that eliminating delayed treatment might rather lead to reduce medical expenses and improve health outcomes.

## Graphical abstract

Comparison of 3‐year survival before and after expansion of health insurance coverage

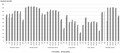

## 1 INTRODUCTION

Cancer incidence and mortality are rapidly growing worldwide, and the global cancer burden has risen to 18.1 million cases and 9.5 million cancer deaths in 2018.^1^, ^2^ Over the last decade, significant strides have been made in the advancement of cancer care, with meaningful impacts on patients.^3^ Earlier detection through testing and advances in chemotherapy and personalized therapies based on tumor biomarkers have led to improvement in cancer survival.^4^, ^5^ Although trends in the United States show that 5‐year relative survival in adults with solid cancer has increased over the last 40 years.^6^, ^7^ cancer remains the second leading cause of death. The prevalence of cancer continues to increase, and expenditures on cancer care are predicted to continue increasing.^8^, ^9^ Moreover, cost‐sharing might lead to bankruptcy of many patients.^3^, ^10^, ^11^

In South Korea, cancer is regarded as one of the most significant health problems,^12^ and cancer has been the leading cause of death since 1983.^12^ In 2018, Korea spent $2.9 billion (4.9% of healthcare expenditures) on cancer patients, who accounted for 0.6% of the total population.^13^ Korea achieved universal health coverage in 1987, and has taken actions by reducing cost‐sharing to ensure financial protection and access to care for everyone.^14^ For cancer patients, the proportion of cost‐sharing has been lowered, from 30% to 10% in 2005 and from 10% to 5% in 2009.^15^, ^16^ Despite novel cancer medicines such as immunotherapies have recently revolutionized the treatment of non‐small cell lung cancer, these medicines are extremely expensive.^17^ Therefore, the government is faced with consideration of financial sustainability and resource allocation as well as patient's access to medicines.

To ensure access to high‐cost medicines, Korean government implemented two policies.^18^ First, alleviation of pricing requirement and managed entry agreement with pharmaceutical companies were introduced for drugs for rare diseases and cancer since May 2013. Basic pricing and reimbursement are based on both a reimbursement decision based on cost‐effectiveness and a price negotiation since 2007.^19^ Second, the government has expanded the reimbursement criteria from the restricted extent to other indications, first‐line therapies, and extra doses among already‐listed medicines in the health insurance benefit package from 2013 to 2016.

It is known that having health insurance reduces out‐of‐pocket of patients, thereby can affect the amount and quality of health care that an individual receives, and thus may be important for patients’ survival.^20^, ^21^, ^22^ There is extensive evidence that aspects of insurance status affect cancer survival, but only a few studies have examined the impacts of enhancing health insurance benefit coverage in medicines on cancer survival.

Cancers of the stomach; the colon and rectum (colorectal); the trachea, bronchus, and lung (lung); thyroid; liver; breast; and prostate impose a substantial burden of disease in Korea as a whole. In 2017, these six common types of cancers except thyroid cancer are expected to account for approximately 58.3% of new cases in Korea.^23^ We investigated the impact of health insurance benefit reimbursement expansion on the healthcare utilization and outcomes of patients with six types of solid cancer in Korea by utilizing the National Health Insurance (NHI) database.

## 2 MATERIALS AND METHODS

### 2.1 Data source

This study compared survival, healthcare utilization, and expenses before and after the policy using health insurance claims data. Newly diagnosed cancer patients aged 18 and over in 2010 and 2013 were followed up for 3 years, and the electronic data of patients treated from January 2009 to December 2015 with complete payments were obtained from medical institutions. Korea's NHI research database contains the registry files and medical benefit claims of 50 million people. The NHI program is a universal healthcare system, in which beneficiaries can access any of the contracted medical facilities and institutions in the country by making a low co‐payment. The NHI research database includes data on all ambulatory claims, inpatient claims, inpatient orders, and prescriptions dispensed at contracted medical institutions and pharmacies.

Patients’ drug history from the included medical institutions and prescribed pharmacy medications were reviewed. Next, their health insurance expenses over time, benefit payments, and the expenses of drugs over time used by inpatients and outpatients were calculated. The diagnostic terms used are based on the International Statistical Classification of Diseases and Related Health Problems, 10th Revision (ICD‐10). From patients’ health insurance claims history, data were extracted on patients’ demographic information, disease codes, characteristics of the visited institution, hospitalization period, medical expenses, out‐of‐pocket expenses, drug components, and medication cost as variables.

In order to examine the effects of the drug coverage enhancement policy on cancer patients, a before‐after study design was used, and newly admitted cancer patients in 2013 and 2010 were compared.

### 2.2 Study population and measures

Patients with six types of malignant solid tumors were defined as those admitted to the hospital with a primary diagnosis (primary disease code) or first secondary diagnosis of the corresponding type of cancer. In order to include new cancer patients, some patients were excluded from the study. In detail, inpatients with a main diagnosis or secondary diagnosis during the last year and outpatients with at least one medical institution visit were excluded from the pre‐policy (Jan 2010 to Dec 2012) patients. Similarly, patients who developed cancer during the post‐policy period (Jan 2013 to Dec 2015) were also excluded. Additionally, patients with overlapping data in the 2010 and 2013 cohorts were also excluded.

The outcome indicators of the newly diagnosed patients were survival, healthcare utilization and expenses, and cost of anti‐cancer drugs were compared in terms of person‐years. In this study, a patient was the unit of analysis, and multiple visits by a patient were counted as one visit. Parts of patients’ personal identification numbers were codified and blocked out to protect their privacy, and the authors were blinded to each patient's full personal identification number. In accordance with the Declaration of Helsinki, IRB approval procedures were followed internally.

### 2.3 Analysis

We estimated the propensity score for policy without regard to outcomes by multiple logistic regression analysis using age category, sex, and stage. Matching was done 1:1 using the Greedy 5→1 digit matching macro with the estimated propensity score.^24^ We compared baseline characteristics between patient pre‐policy and post‐policy and analyzed differences using the chi‐square test.

We calculated the overall survival per person years by dividing the number of survived patients by the total number of person years. Overall survival was first examined using the Kaplan–Meier method. The crude survival proportion refers to the estimated probability of survival to the end of a period, regardless of cause of death.

We used matched Cox regression models to estimate hazard ratios and their 95% confidence intervals for survival in the propensity‐based matched cohort. By using these models, we could obtain an unbiased estimate of the change in the hazard of mortality. We also did a subgroup analysis according to age category, sex, and ant‐cancer medication use by cancer site, with a significance level of *p* < 0.05. All statistical analyses were performed using SAS version 9.0 (SAS Institute, Cary, NC, USA).

## 3 RESULTS

### 3.1 General characteristics

**FIGURE 1:**
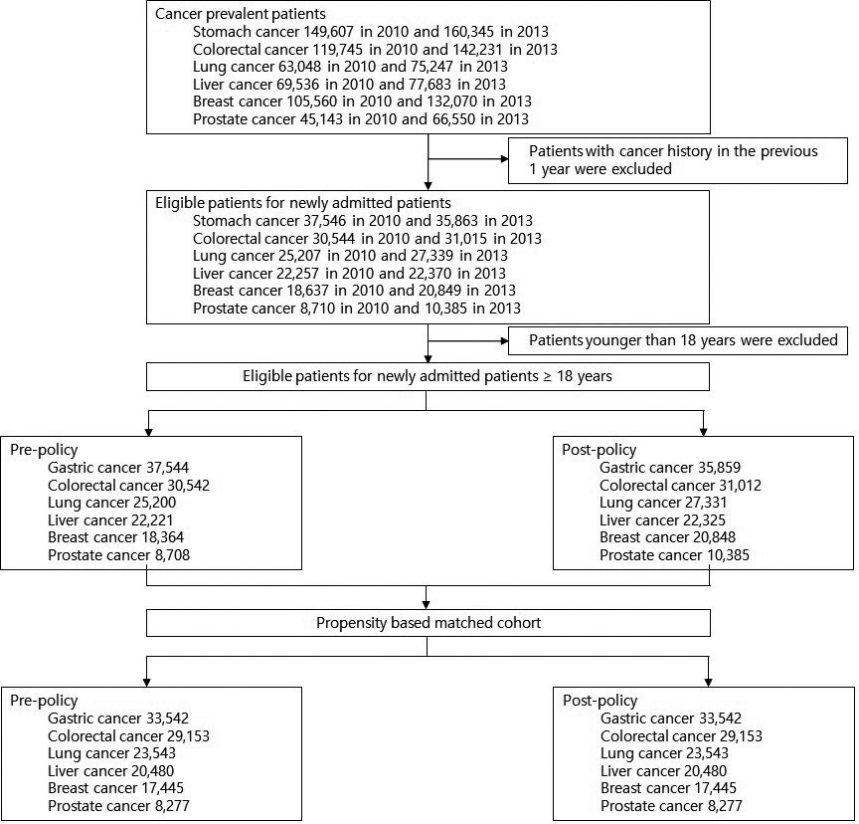
Flow diagram of study cohort selection

A total of 141,037 patients in pre‐polity and 147,760 patients in post‐policy met the study inclusion criteria. After propensity score estimation and matching in a one to one ratio, the cohort used in the analysis included 141,037 in pre‐policy and 141,037 in post‐policy. Figure 1 shows the cohort selection process of newly admitted cancer patients before 2010 and after 2013.

Table 1 shows the baseline characteristics of people in the overall cohort and propensity‐based matched cohort. In the overall cohort, only the number of stomach cancer patients decreased from 37,544 pre‐policy to 35,859 post‐policy. The number of cancer patients rose after the policy, and especially breast and prostate cancer patients showed considerable growth. For five of the six cancer types, patients aged 60 years and over represented the largest age group; however, for breast cancer patients, there were higher proportions in the 40–49‐year and 50–59‐year age groups. The post‐policy patients were older than the pre‐policy patients in the overall cohort, and similar or older than pre‐policy in the matched cohort. More than half of the patients were men for four of the cancers.

**TABLE 1:** General characteristics of patient between pre‐policy and post‐policy

	Overall cohort			Matched by age, gender, stage		
	Pre‐policy	Post‐policy	*p* value	Pre‐policy	Post‐policy	*p* value
Stomach cancer (No. of patients)	37,544	35,859		33,542	33,542	
Male (%)	66.9	67.7	0.0337	67.0	66.2	0.0294
Age (Mean ± SD, years)*	63.4 ± 12.7	64.4 ± 12.6	<0.0001	64.2 ± 12.6	64.1 ± 12.7	0.0514
Stage (%)			<0.0001			<0.0001
0,1	24.3	18.5		20.3	19.7	
2	6.0	5.9		6.7	6.3	
3	5.3	4.8		5.8	5.2	
4	11.9	7.8		8.4	8.4	
Colorectal cancer (No. of patients)	30,542	31,012		29,153	29,153	
Male (%)	59.4	59.4	0.9138	59.3	59.7	0.3277
Age (Mean ± SD, years)*	64.8 ± 12.4	65.7 ± 12.6	<0.0001	65.1 ± 12.3	65.1 ± 12.5	0.0059
Stage (%)			<0.0001			<0.0001
0,1	10.2	9.1		9.05	8.84	
2	8.7	7.4		8.6	7.91	
3	11.1	10.0		11.47	10.62	
4	10.9	8.4		8.94	8.94	
Lung cancer (No. of patients)	25,200	27,331		23,543	23,543	
Male (%)	69.7	68.8	0.0275	69.64	69.51	0.7562
Age (Mean ± SD, years)*	68 ± 11.2	68.6 ± 11.3	<0.0001	68.4 ± 11.1	68.4 ± 11.2	0.0729
Stage (%)			<0.0001			0.3815
0,1	8.5	7.9		8.1	8.3	
2	4.0	3.9		4.3	4.6	
3	10.7	7.7		9.0	8.9	
4	25.7	20.2		24.0	23.5	
Liver cancer (No. of patients)	22,221	22,325		20,480	20,480	
Male (%)	73.1	73.3	0.6855	73.09	73.42	0.4478
Age (Mean ± SD, years)	62.5 ± 12.1	63.7 ± 12.1	<0.0001	62.9 ± 12.0	63.0 ± 12.0	0.9291
Stage (%)			<0.0001			0.3154
0,1	11.8	10.2		11.7	11.2	
2	8.2	5.9		6.4	6.4	
3	6.5	4.1		4.4	4.4	
4	10.4	8.0		8.9	8.7	
Breast cancer (No. of patients)	18,634	20,848		17,445	17,445	
Age (Mean ± SD, years)*	51.6 ± 11.4	52.3 ± 11.4	<0.0001	52.0 ± 11.4	52.1 ± 11.5	0.4832
Stage (%)			<0.0001			0.3155
0,1	24.0	20.2		23.7	24.0	
2	17.7	15.7		18.5	18.7	
3	10.4	7.3		8.7	8.7	
4	8.6	5.6		7.3	6.7	
Unclassified	39.2	51.3		41.9	41.9	
Prostate cancer (No. of patients)	8,708	10,385		8,277	8,277	
Age (Mean ± SD, years)*	69.9 ± 8.9	70.3 ± 8.9	0.0002	70.1 ± 8.9	70.1 ± 8.9	0.6056
Stage (%)			<0.0001			0.9999
0,1	5.6	3.0		3.3	3.3	
2	9.4	6.4		8.1	8.1	
3	4.2	3.6		3.8	3.8	
4	4.8	3.2		4.0	3.9	

* For variables are shown as percentages. Plus‐minus values are means ± SD.

### 3.2 Healthcare utilization

Table 2 shows the healthcare utilization. The average numbers of days of hospitalization and outpatient visits per person increased after the policy. However, for stomach cancer, the in‐hospital length of stay (LOS) decreased slightly and outpatient visits declined for colorectal and lung cancer. The in‐hospital LOS was the longest (58.0 and 57.5 days before and after the policy, respectively) for lung cancer, and the number of outpatient visit days per patient was the highest (57.9 and 60.2 days before and after the policy, respectively) for breast cancer.

**TABLE 2:** The absolute change in healthcare utilization and spending

	Stomach cancer		Colorectal cancer		Lung cancer		Liver cancer		Breast cancer		Prostate cancer	
	Pre‐policy	Post‐policy	Pre‐policy	Post‐policy	Pre‐policy	Post‐policy	Pre‐policy	Post‐policy	Pre‐policy	Post‐policy	Pre‐policy	Post‐policy
Utilization												
Length of stay in hospital per patient (days)	40.1 ± 70.5	39.6 ± 77.2	52.5 ± 85.0	54.1 ± 93.9	58.0 ± 77.5	57.5 ± 81.8	47.5 ± 63.5	48.2 ± 70.0	45.7 ± 80.7	53.8 ± 101.8	31.0 ± 77.3	33.9 ± 91.2
Outpatient visits per patient (days)	21.1 ± 19.4	20.8 ± 18.6	29.7 ± 28.4	28.6 ± 28.2	29.0 ± 29.9	28.6 ± 29.9	24.3 ± 21.9	24.5 ±24.6	57.9 ± 38.0	60.2 ±38.8	28.0 ± 24.0	27.0 ± 23.6
Expenditure												
Medical expenditure per patient (thousand USD)	9.2	9.6	13.7	13.8	15.2	14.9	13.8	14.8	16.6	17.6	7.6	8.2
Medical costs in inpatient per patient (thousand USD)	7.2	7.2	10.3	10.6	11.3	11.2	11.0	11.7	7.8	7.9	4.5	4.7
Medical costs in outpatient per patient (thousand USD)	1.9	2.1	3.3	3.3	3.9	3.7	2.8	3.1	8.8	9.6	3.1	3.5
Anti‐cancer therapy												
No. of changes in anti‐cancer drugs	4.6 ± 4.9	4.4 ± 4.7	4.9 ± 5.1	5.1 ± 5.3	6.0 ± 5.5	6.2 ± 5.7	3.4 ± 3.4	4.0 ± 4.3	5.9 ± 5.7	6.7 ± 6.2	4.6 ± 5.4	4.7 ± 5.7
Anti‐cancer drug costs per patient (thousand USD)	2.7	2.9	4.1	3.4	5.1	4.7	0.7	1.4	7.1	6.7	3.3	3.2
Anti‐cancer drug costs in inpatient per patient (thousand USD)	1.3	1.1	2.4	2.1	2.2	1.6	0.3	0.4	2.2	1.8	0.5	0.4
Anti‐cancer drug costs in outpatient visit per patient (thousand USD)	1.4	1.8	1.8	1.3	2.9	3.1	0.4	1.1	4.8	4.9	2.8	2.8

Note

Plus‐minus values are means ± SD.

Medical expenditures per patient, including inpatient and outpatient care, increased for all types of cancer, except for lung cancer. For breast cancer, total medical expenditures were the highest ($16,600 and $17,600 before and after the policy, respectively).

The frequency of switching to a different anti‐cancer drug showed similar patterns before and after the policy. For breast cancer, the number of drug changes was the highest, and increased most drastically, from 5.9 times pre‐policy to 6.7 times post‐policy. For stomach cancer and colorectal cancer, the number of anti‐cancer drug changes decreased after the policy. The cost of anti‐cancer drugs per patient was the highest for patients with breast cancer followed by lung cancer. The total cost of anti‐cancer drugs decreased for five cancer sites, and anti‐cancer drug expenditure increased only in liver cancer.

### 3.3 Overall survival

**FIGURE 2:**
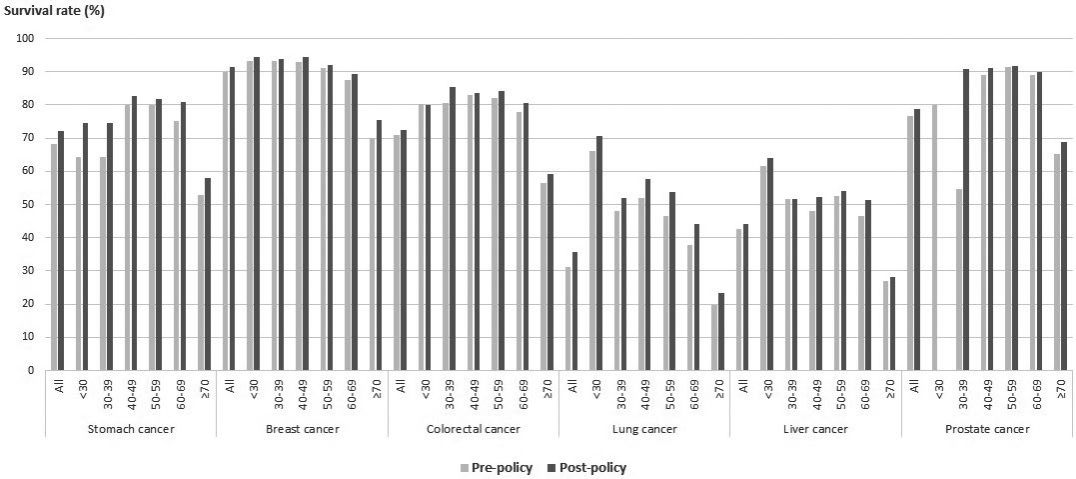
3‐year survival by age

The overall survival for post‐policy patients with all six cancers was higher than that for pre‐policy patients. For each of the six cancers, the 3‐year survival rate of breast cancer patients was the highest. And lung cancer patients showed the lowest survival among the six cancers, but they showed the most notable improvement in their survival compared to pre‐policy patients (Figure 2).

After multivariable adjustment in the matched cohort, the expansion policy of health insurance benefit reimbursement was associated with improved survival in five cancers except for breast cancer in comparison to before the policy In the multivariate Cox model, patients with stomach cancer (hazard ratio [HR] 0.88, 95% confidence interval [CI] 0.86–0.91, *p* < 0.0001), colorectal cancer (HR 0.90, 95% CI 0.87–0.93, *p* < 0.0001), lung cancer (HR 0.87, 95% CI 0.86–0.89, *p* < 0.0001), liver cancer (HR 0.91, 95% CI 0.89–0.94, *p* < 0.0001), and prostate cancer (HR 0.89, 95% CI 0.83–0.94, *p* < 0.0001) had significantly higher survival rates post‐policy than pre‐policy in propensity score‐matched cohort. Older age, male, anti‐cancer drug use (except breast cancer), and higher stage (except prostate cancer) were all independently associated with lower OS (Table 3).

**TABLE 3:** Cox‐proportional hazards for all‐cause mortality

Factor	Overall cohort			Matched cohort		
	HR	95% CI	*p* value	HR	95% CI	*p* value
Stomach cancer	0.89	(0.87–0.92)	<0.0001	0.88	(0.86–0.91)	<0.0001
Breast cancer	0.94	(0.88–1.00)	0.0416	0.96	(0.89–1.02)	0.2062
Colorectal cancer	0.91	(0.88–0.93)	<0.0001	0.90	(0.87–0.93)	<0.0001
Lung cancer	0.87	(0.85–0.88)	<0.0001	0.87	(0.86–0.89)	<0.0001
Liver cancer	0.91	(0.89–0.94)	<0.0001	0.91	(0.89–0.94)	<0.0001
Prostate cancer	0.87	(0.82–0.93)	<0.0001	0.89	(0.83–0.94)	0.0002

Note

We adjusted age, gender, anti‐cancer drug use, and stage of cancer.

Abbreviations: CI, Confidence interval; HR, hazard ratios.

## 4 DISCUSSION

In 2013, the Korean government enhanced coverage for cancer by increasing reimbursement for new medicines and has expanded the drug benefit reimbursement to other indications or extra doses for the already‐listed drugs for 3 years. Examples of the expansion of health insurance coverage for medicines include adding a single use for soft tissue sarcoma, biweekly therapy for prostate cancer, and indications. For example, we have expanded the indications of health insurance coverage everolimus for postmenopausal advanced breast cancer or trastuzumab for neoadjuvant in breast cancer. Also, we allowed for patients to use medicines as first‐line treatment those medicines which were only not covered in health insurance in the past such as cetuximab biweekly therapy as first‐line treatment in colorectal cancer.

This study is one of few studies that have analyzed survival outcomes after expansion of benefit coverage. We found that post‐policy patients in stomach, colorectal, lung, liver, breast, and prostate cancers have better survival as well as healthcare expenditure and drug costs increased slightly. It is believed that first‐line use of target drugs might eliminate delayed treatment, thereby timely treatment might rather lead to increase anti‐cancer use, medical expenditure and improve health outcomes such as survival.

Our results are similar to those of previous studies showing that cancer patients with health insurance had better outcomes in the United States.^20^, ^21^, ^22^, ^25^, ^26^, ^27^, ^28^, ^29^, ^30^, ^31^, ^32^, ^33^, ^34^ Previous studies have focused on the effects of health insurance on patients’ health. Parikh et al. (2014) investigated the impact of health insurance status on survival among colorectal cancer patients using the Tennessee cancer registry, and found that uninsured patients had worse overall survival.^22^ Cole AP et al. (2019) examined association insurance and ovarian, pancreatic, lung, colorectal, prostate, and breast cancer survival.^25^ Amini et al. (2015) reported that insured melanoma patients had a more favorable overall survival rate.^26^ Many studies have investigated the effects of introducing Medicare part D, including studies of generic medicine use, emergency department visits, out‐of‐pocket payments made by patients, reduced inequality in prescriptions, and hospitalization rates.^27^, ^28^, ^29^, ^30^, ^31^, ^32^, ^33^, ^34^ Healthcare utilization of previous studies is similar. Kim et al. (2014) reported that reducing out‐of‐pocket of cancer patients led to an increase in the utilization of outpatient services across all income group.^15^ Loehrer et al. (2016) examined the 2006 Massachusetts healthcare reform, a model for the Affordable Care Act, was associated with access to care for patients with colorectal cancer.^35^

To the best of our knowledge, this is the first population‐based study focusing on the overall survival associated with expanding drug reimbursement for already‐listed medicines. Most existing studies were limited to specific states, whereas this study included all patients with incident cancer in the total population of the nation; therefore, the results provide useful evidence for the health insurance policy.

The findings of this study provide insights into the design of health insurance benefits packages. We compared the outcomes of interest using a quasi‐experimental design and evaluated the effects of the coverage expansion policy on patients’ health. Thus, these findings may furnish meaningful evidence for designing health insurance systems in other countries. A strength of this study is that it obtained real‐world, generalizable information by using health insurance claims data from the total population. As outcome measures, this study examined patients’ survival. Furthermore, the in‐hospital LOS, outpatient visits, total medication expenses including outpatient visits, and drug costs were also evaluated. Previous studies on the impacts of this policy have used medical utilization, equity, overburdened medical expenditure, and the mortality rate as result indicators. We have analyzed direct estimates of access to care defined as medical utilization, expenditures, and drug costs as well as the final outcomes of survival.

However, this study has the following limitations. As a retrospective study utilizing an administrative dataset, the variables used in this analysis were limited therefore, there may be several important confounding variables that we could not control such as laboratory data. In addition, the details of patients’ health conditions as well as complications of surgical and adjuvant therapy were not known. Also, this study has no control group, so we cannot adjust many unmeasured confounders. It is a limitation that we could not distinguish whether improvement in survival was the effect of policy or other factors. Given the observational study design, we did not attempt to control for other policies introduced during the same time frame. A simple comparison between pre‐ and post‐policy implementation cannot control for non‐policy implementation‐related variations over time, so this may restrict the ability to evaluate the net effects of a policy, and the endogenous or missing variables lead to bias. Our findings are subject to selection bias and confounding in the baseline between before and after. We tried to eliminate the baseline difference using propensity score matching, which might be greater than stratification or covariate adjustment. However, other bias may remain because of unmeasured confounders.

This study evaluated only all‐cause mortality, and other outcomes such as progression‐free survival are unknown. Changes in the survival rate may also be attributed to the effects of other factors, such as developments in healthcare technology and changes in patients’ health conditions. In this respect, larger studies linking health insurance data with electronic medical records are needed.

In summary, the enhancement of drug coverage for cancer patients led to a slight decrease in inpatient medical expenses and drug expenses per patient, while the outpatient expenses per capita increased. This policy change also contributed to the increased survival rate of cancer patients. Although the increased usage of anti‐cancer drugs significantly contributed to the survival of cancer patients, the problem of financial sustainability emerges due to patients’ overestimation of potential medication benefits and the introduction of new, high‐priced anti‐cancer medications. Further research should investigate ways to explain which therapies contributed to better OS, and to ensure value‐based anti‐cancer drug treatments.

## CONFLICT OF INTEREST

All authors report there are no conflicts of interest.

## ETHICS STATEMENT

This study was approved by the institutional review board (IRB No. HIRA2017‐014–001).

## Data Availability

Research data are not shared.
